# Vital Signs: Overdoses of Prescription Opioid Pain Relievers and Other Drugs Among Women — United States, 1999–2010

**Published:** 2013-07-05

**Authors:** Karin A. Mack, Christopher M. Jones, Leonard J. Paulozzi

**Affiliations:** Div of Unintentional Injury Prevention, National Center for Injury Prevention and Control, CDC

## Abstract

**Background:**

Overdose deaths have increased steadily over the past decade. This report describes drug-related deaths and emergency department (ED) visits among women.

**Methods:**

CDC analyzed rates of fatal drug overdoses and drug misuse- or abuse-related ED visits among women using data from the National Vital Statistics System (1999–2010) and the Drug Abuse Warning Network (2004–2010).

**Results:**

In 2010, a total of 15,323 deaths among women were attributed to drug overdose, a rate of 9.8 per 100,000 population. Deaths from opioid pain relievers (OPRs) increased fivefold between 1999 and 2010 for women; OPR deaths among men increased 3.6 times. In 2010, there were 943,365 ED visits by women for drug misuse or abuse. The highest ED visit rates were for cocaine or heroin (147.2 per 100,000 population), benzodiazepines (134.6), and OPR (129.6). ED visits related to misuse or abuse of OPR among women more than doubled between 2004 and 2010.

**Conclusions:**

Although more men die from drug overdoses than women, the percentage increase in deaths since 1999 is greater among women. More women have died each year from drug overdoses than from motor vehicle–related injuries since 2007. Deaths and ED visits related to OPR continue to increase among women. The prominent involvement of psychotherapeutic drugs, such as benzodiazepines, among overdoses provides insight for prevention opportunities.

**Implications for Public Health Practice:**

Health-care providers should follow guidelines for responsible prescribing, including screening and monitoring for substance abuse and mental health problems, when prescribing OPR. Health-care providers who treat women for pain should use their state’s prescription drug monitoring program and regularly screen patients for psychological disorders and use of psychotherapeutic drugs, with or without a prescription.

## Introduction

In 2010, enough opioid pain relievers (OPR) were sold to medicate every adult in the United States with the equivalent of a typical dose of 5 mg of hydrocodone every 4 hours for 1 month (1), a 300% increase in the sales rate over 11 years. This rise in distribution of OPR is concomitant with increasing rates of drug overdose death and chronic, nonmedical use of OPR (2,3).

Differences between men and women related to prescription drug use outcomes are complicated. The death rate for OPR overdose is higher among men than women, but since 1993, hospitalizations for OPR overdoses have been more frequent among women than men (4). During 2004–2008, women and men had similar emergency department (ED) visit rates related to nonmedical use of OPR and benzodiazepines (5). OPR prescribing and use patterns also differ by gender. Women are more likely than men to be prescribed OPR, to use them chronically, and to receive prescriptions for higher doses of OPR (6,7). This might be because the most common forms of pain are more prevalent among women, and pain is more intense and of longer duration in women than men (8,9). Women also might be more likely than men to engage in “doctor shopping” (receiving a prescription for a controlled substance from multiple providers), and more likely to be prescribed OPR combined with sedatives (10,11). Sex-specific health risks associated with long-term OPR use among women include amenorrhea and infertility (12,13). Finally, the progression to dependence on OPR might be accelerated in women, and women with substance use disorders are more likely than men to face barriers in access to substance abuse treatment (14,15). Taken together, these health concerns indicate a need to examine drug overdose deaths and ED visits among women to guide development of targeted prevention strategies.

## Methods

**FIGURE 1:**
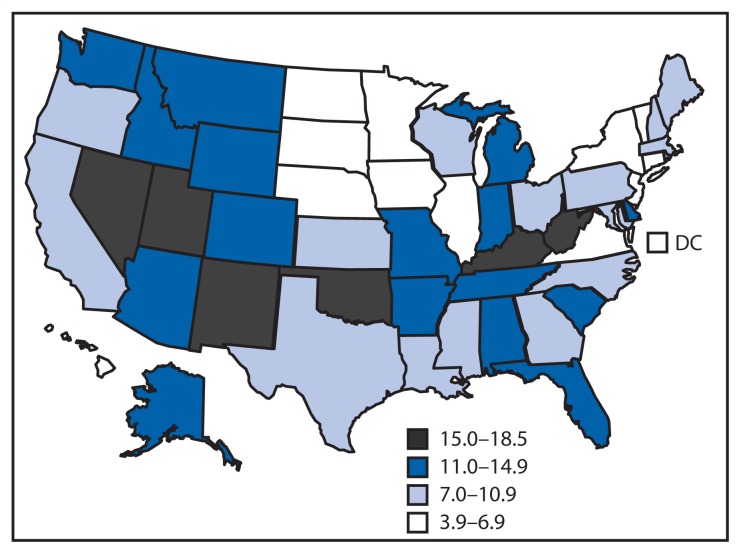
Age-adjusted death rates* for drug overdose deaths among women — National Vital Statistics System, United States, 2009–2010 * Deaths per 100,000 population; age-adjusted to the 2000 U.S. standard population using the bridge-race estimates.

For this report, death rates are based on the National Vital Statistics System multiple cause of death files (1999–2010). Drug poisoning deaths, referred to as drug overdose deaths in this report, were defined as those with an underlying cause of death classified using the *International Classification of Diseases, 10th Revision* (ICD-10) external cause of injury codes as X40-X44, X60-X64, X85, or Y10-Y14. Rates include injury deaths of any intent (unintentional, suicide, homicide, or undetermined) for U.S. residents. Among deaths with drug overdose as the underlying cause, CDC identified the type of drug involved based on ICD-10 codes for prescription drugs (T36-T39, T40.2-T40.4, T41-T43.5, and T43.8-T50.8), prescription OPR (T40.2-T40.4), benzodiazepines (T42.4), antidepressants (T43.0-T43.2), heroin (T40.1), and cocaine (T40.5). The codes used to categorize prescription drugs might capture some over-the-counter medications. Deaths involving more than one type of drug were counted in multiple categories. Rates were age adjusted to the 2000 U.S. Census population using bridged-race population estimates (Figure 1).*

The Substance Abuse and Mental Health Services Administration’s Drug Abuse Warning Network (DAWN) is a public health information system that tracks the impact of drug use, misuse, and abuse in the United States by monitoring drug-related hospital ED visits. This report used 2004–2010 DAWN public use files for analyses.† DAWN collects data from a stratified, simple random sample of approximately 220 nonfederal, short-stay general hospitals that operate 24-hour EDs. Rates presented in this report are based on the numbers of ED visits weighted to be representative of the U.S. population. Denominators for this report were based on U.S. Census postcensal estimates. DAWN defines misuse or abuse of a drug, based on information in the medical record, as taking a higher-than-recommended dose, taking a drug prescribed for another person, drug-facilitated assault (patient was administered a drug by another person for a malicious purpose), or documented misuse or abuse. ED visits related to the misuse or abuse of alcohol only by persons aged <21 years, which are typically included in DAWN misuse or abuse estimates, were not included in this analysis. ED visits involving more than one type of drug were counted in multiple categories.

## Results

In 2010, a total of 15,323 deaths among women were attributed to drug overdose, a rate of 9.8 per 100,000 population. Among these, a drug was specified in 10,922 (71.3%) deaths. One or more prescription drugs were involved in 9,292 (85%) of the drug-specified deaths among women, and OPRs were involved in 6,631 (71.3%) of the prescription drug overdose deaths. These numbers represent substantial increases from 1999 (5,591 drug overdose deaths among women and 1,287 OPR overdose deaths). The percentage increase in number of OPR overdose deaths was 415% for women and 265% for men. The rate for OPR deaths (4.2 per 100,000 population) was four times the rate for cocaine and heroin deaths combined (1.0) (Table 1). The drug overdose death rate among men (23,006 drug overdoses and 10,020 OPR overdose deaths in 2010) was 1.55 times the rate among women for all drugs (down from 2.1 times the rate in 1999).

Death rates varied by age and race. The rate for all drug overdose deaths among women was highest among those aged 45–54 years (21.8 per 100,000 population). American Indian/Alaska Native (14.5) and non-Hispanic white (12.7) women had the highest drug overdose death rates. The rate of suicide drug overdose deaths was similar for women (1.8) and men (1.7), although drug overdose–related suicide deaths accounted for 34% of all suicide deaths among women compared with 8% among men. OPRs were involved in one in 10 suicides among women.

In 2010, women made 943,365 ED visits for drug misuse or abuse; a rate of 601 per 100,000 population (Table 2) (for every OPR overdose death there were 30 ED visits for OPR misuse or abuse). Cocaine or heroin (147.2), benzodiazepines (134.6), and OPR (129.6) were associated with the highest ED visit rates. ED visit rates among women for all drugs tended to be highest among those aged 25–34 years. The rates for all drug or OPR misuse- or abuse-related ED visits were not significantly different between men and women. The all drug rate for men was 1.35 times the rate for women in 2010, and the OPR rate for men was 1.2 times the rate for women.

During 2009–2010, rates for drug overdose deaths among women varied widely by state (Figure 1). Age-adjusted drug overdose death rates ranged from 3.9 per 100,000 women in North Dakota to 18.5 in Nevada.

**FIGURE 2:**
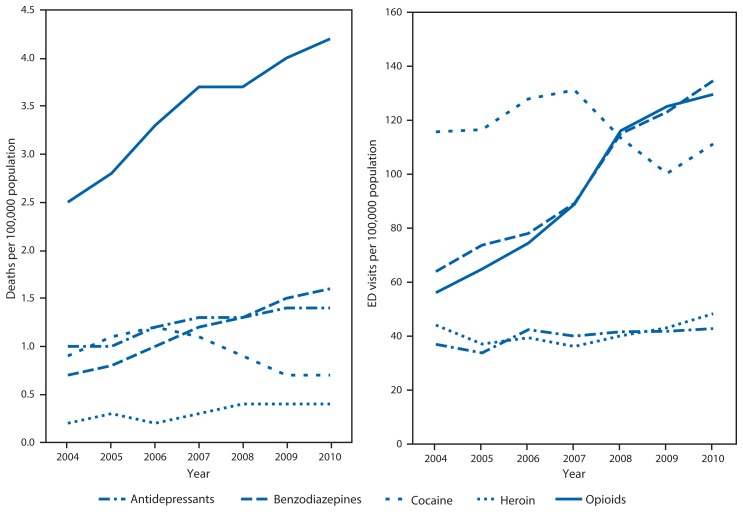
Crude rates* for drug overdose deaths and drug misuse- or abuse-related emergency department (ED) visits among women, by select drug class — National Vital Statistics System and Drug Abuse Warning Network, United States, 2004–2010 * Scales differ for deaths and emergency department visits.

During 2004–10, OPR death rates and ED visit rates increased substantially among women (Figure 2). During this period, the rate of OPR deaths among women increased 70% and the rate of OPR misuse- or abuse-related ED visits more than doubled. Cocaine deaths and ED visits declined during the same period. Starting in 2008, more women visited EDs because of misuse or abuse of benzodiazepines or OPR than for cocaine.

## Conclusions and Comment

Since 2007, more women have died from drug overdoses than from motor vehicle traffic injuries, and in 2010, four times as many died as a result of drug overdose as were victims of homicide. Men are more likely than women to die from drug overdose; however, between 1999 and 2010, the percentage increase in the rate of overdose deaths was greater for women (151%) than for men (85%). The prescribing of controlled substances, drug overdose deaths, and drug misuse- and abuse-related ED visits among women have risen despite numerous recommendations over the past decade for more cautious use of OPR and efforts to curb abuse and prevent deaths.

Between 1999 and 2010, OPR overdose deaths increased more than fivefold among women (a total of 47,935 OPR overdose deaths during that period). Abuse of OPR is a particular problem for women of childbearing age. Given the risk for neonatal abstinence syndrome as a result of OPR abuse during pregnancy (16), and the potential effects of OPR on an embryo during the first trimester (17), health-care providers should include discussions of pregnancy plans within the context of treatment and monitoring of patients taking OPR for medical or nonmedical reasons. Women treated for OPR abuse should be counseled regarding risks to the fetus of OPR abuse during pregnancy. The risks and benefits of treatment of chronic conditions with OPR during pregnancy should be weighed carefully (18). Use of benzodiazepines and antidepressants during pregnancy, or at any time in combination with OPR, also should be considered carefully by women and their health-care providers. Psychological conditions, which might co-occurr with pain or substance abuse (19)*,* need to be assessed and addressed within a treatment regime.

The findings in this report are subject to at least four limitations. First, vital statistics underestimate the rates of drug involvement in deaths because the type of drug is not specified on many death certificates. Second, injury mortality data might underestimate by up to 35% the actual numbers of deaths for American Indian/Alaskan Natives and certain other racial/ethnic populations (e.g., Hispanics) because of the misclassification of race/ethnicity of decedents on death certificates (20). Third, all the drugs involved in ED visits might not be identified. Fourth, information on the motivation for use might be incomplete; some ED visits might have resulted from suicide attempts. Finally, distinguishing between drugs taken for nonmedical and medical reasons is not always possible, especially when multiple drugs are involved.

Public health interventions to reduce prescription drug overdose must strike a balance between reducing misuse and abuse and safeguarding legitimate access to treatment. Health-care providers who treat women for pain should follow prescribing guidelines. Providers should screen all their patients for psychological disorders and for use of psychotherapeutic drugs, either with or without a prescription. Checking state prescription drug monitoring programs before long-term prescribing of controlled substances should be a standard of care. Communities should try to increase access for women, especially pregnant women, to substance abuse treatment services. Medicaid programs, which enroll disproportionate numbers of young women, should ensure that the prescribing of controlled substances to their clients meets established guidelines. Overdose deaths and ED visits related to prescription drugs, especially OPR, continue to be unacceptably high, and targeted efforts are needed to reduce the number of deaths in this epidemic.

## Figures and Tables

**TABLE 1:** Drug overdose deaths* and rates† among women, by selected characteristics, and comparison with 1999 — National Vital Statistics System, United States, 2010

Characteristic	Antidepressants		Benzodiazepines		Cocaine/Heroin		Opioids		All prescription drugs		All drugs		M:F rate ratio (all drugs), 2010	% change in female rate (all drugs), 1999 to 2010
					
	No.	Rate	No.	Rate	No.	Rate	No.	Rate	**No.**	Rate	No.	Rate		
					
		(CI)		(CI)		(CI)		(CI)		(CI)		(CI)		
**Total**	**2,204**	**1.4 (1.3–1.5)**	**2,579**	**1.6 (1.6–1.7)**	**1,598**	**1.0 (1.0–1.1)**	**6,631**	**4.2 (4.1–4.3)**	**9,292**	**5.9 (5.8–6.0)**	**15,323**	**9.8 (9.6–9.9)**	**1.55**	**151.3**
**Age groups (yrs)**														
<18	12	—	15	—	11	—	66	0.2 (0.1–0.2)	91	0.3 (0.2–0.3)	138	0.4 (0.3–0.4)	1.50	100.0
18–24	66	0.4 (0.3–0.6)	159	1.1 (0.9–1.2)	172	1.1 (1.0–1.3)	396	2.6 (2.4–2.9)	511	3.4 (3.1–3.7)	899	6.0 (5.6–6.4)	2.58	160.9
25–34	285	1.4 (1.2–1.6)	451	2.2 (2.0–2.4)	326	1.6 (1.4–1.8)	1,093	5.3 (5.0–5.7)	1,423	7.0 (6.6–7.3)	2,422	11.9 (11.4–12.3)	2.10	158.7
35–44	483	2.3 (2.1–2.5)	593	2.9 (2.6–3.1)	381	1.8 (1.7–2.0)	1,515	7.3 (7.0–7.7)	2,014	9.8 (9.3–10.2)	3,464	16.8 (16.2–17.3)	1.48	93.1
45–54	785	3.4 (3.2–3.7)	839	3.7 (3.4–3.9)	526	2.3 (2.1–2.5)	2,239	9.8 (9.4–10.2)	2,986	13.1 (12.6–13.5)	4,986	21.8 (21.2–22.4)	1.31	202.8
55–64	452	2.0 (2.2–2.6)	386	2.0 (1.8–2.2)	166	0.9 (0.7–1.0)	1,038	5.5 (5.2–5.8)	1,530	8.1 (7.7–8.5)	2,436	12.9 (12.4–13.4)	1.34	268.6
≥65	121	0.5 (0.4–0.6)	136	0.6 (0.5–0.7)	15	—	284	1.2 (1.1–1.4)	737	3.2 (3.0–3.4)	977	4.3 (4.0–4.5)	1.00	65.4
**Race/Ethnicity** §														
White	1,907	1.9 (1.8–2.0)	2,320	2.3 (2.2–2.4)	1,015	1.0 (0.9–1.1)	5,757	5.7 (5.5–5.8)	7,990	7.9 (7.8–8.0)	12,946	12.7 (12.5–12.9)	1.50	188.6
Black	148	0.7 (0.6–0.8)	117	0.6 (0.5–0.7)	414	2.0 (1.8–2.2)	425	2.1 (1.9–2.3)	635	3.1 (2.8–3.3)	1,217	5.9 (5.6–6.2)	1.71	55.3
American Indian/Alaska Native	25	1.9 (1.2–2.8)	21	1.6 (1.0–2.5)	22	1.7 (1.1–2.5)	96	7.3 (5.9–9.0)	120	9.2 (7.5–10.8)	190	14.5 (12.5–16.6)	1.26	190.0
Asian/Pacific Islander	16	—	13	—	11	—	40	0.5 (0.3–0.6)	87	1.0 (0.8–1.3)	126	1.5 (1.2–1.8)	1.67	50.0
Hispanic	99	0.4 (0.3–0.5)	99	0.4 (0.3–0.5)	127	0.5 (0.4–0.6)	294	1.2 (1.0–1.3)	436	1.9 (1.7–2.1)	794	3.2 (3.0–3.4)	2.19	68.4
**Intent**														
Unintentional	1,349	0.9 (0.8–0.9)	1,938	1.2 (1.2–1.3)	1,457	0.9 (0.9–1.0)	5,144	3.3 (3.2–3.4)	6,483	4.1 (4.0–4.2)	11,168	7.1 (4.0–4.2)	1.75	238.1
Suicide	624	0.4 (0.4–0.4)	451	0.3 (0.3–0.3)	52	0.0 (0.0–0.0)	820	0.5 (0.5–0.6)	1,887	1.2 (1.1–1.3)	2,748	1.8 (1.7–1.8)	0.94	50.0
Undetermined	231	0.1 (0.1–0.2)	189	0.1 (0.1–0.1)	87	0.1 (0.0–0.1)	658	0.4 (0.4–0.5)	908	0.6 (0.5–0.6)	1,385	0.9 (0.8–0.9)	1.11	50.0

**Abbreviations:** CI = confidence interval; M:F = male to female.

* Drug-related homicide deaths are not included as a separate row because of small numbers, but are included in overall numbers. Deaths involving more than one type of drug were counted in multiple categories.

† Per 100,000 population.

§ Persons identified as Hispanic might be of any race. Persons identified as any of the other categories were non-Hispanic.

**TABLE 2:** Drug misuse- or abuse-related emergency department visits among women, by selected characteristics and rates,* and comparison with 2004 — Drug Abuse Warning Network, United States, 2010

Characteristic	Antidepressants		Benzodiazepines		Cocaine/Heroin		Opioids		All prescription drugs		All drugs		M:F rate ratio (all drugs), 2010	% change (all drugs), 2004 to 2010
					
	No.	Rate	No.	Rate	No.	Rate	No.	Rate	No.	Rate	No.	Rate		
					
		(CI)		(CI)		(CI)		(CI)		(CI)		(CI)		
**Total**	**67,151**	**42.8 (33.2–52.4)**	**211,339**	**134.6 (97.4–171.9)**	**231,058**	**147.2 (100.8–193.6)**	**203,417**	**129.6 (98.7–160.4)**	**672,049**	**428.2 (333.8–522.5)**	**943,365**	**601.0 (472.0–730.1)**	**1.35**	**47.0**
**Age groups (yrs)**														
<18	4,013	11.1 (5.9–16.2)	4,379	12.1 (6.4–17.8)	4,332	12.0 (6.0–17.9)	5,351	14.8 (9.8–19.7)	45,166	124.6 (88.5–160.8)	66,353	183.1 (132.6–233.6)	0.99	5.0
18–24	9,914	66.0 (42.8–89.3)	29,446	196.2 (130.2–262.2)	33,841	225.4 (163.0–287.9)	30,719	204.6 (130.6–278.6)	104,691	697.5 (538.1–856.8)	159,189	1,060.0 (830.6–1,290.4)	1.37	45.0†
25–34	15,368	75.2 (57.6–92.8)	57,262	280.3 (167.7–392.8)	65,405	320.1 (213.3–426.9)	47,246	231.2 (163.5–298.9)	154,672	757.0 (547.6–966.4)	225,190	1,102.2 (824.9–1,379.4)	1.31	47.4
35–44	14,224	68.9 (52.8–85.1)	46,314	224.5 (157.8–291.1)	60,866	295.0 (197.4–392.5)	41,558	201.4 (146.3–256.5)	128,086	620.7 (468.4–773.1)	188,304	912.6 (701.0–1,124.2)	1.31	33.2
45–54	15,301	66.9 (47.0–86.8)	43,457	190.1 (146.8–233.3)	52,035	227.6 (134.5–320.6)	43,860	191.8 (143.2–240.4)	128,633	562.6 (438.2–687.0)	179,531	785.2 (608.8–961.6)	1.46	74.0†
55–64	5,481	29.0 (19.9–38.2)	19,676	104.2 (68.4–140.0)	13,776	73.0 (27.7–118.2)	19,761	104.7 (79.6–129.7)	57,580	305.0 (232.9–377.0)	71,132	376.7 (287.7–465.8)	1.53	142.7†
≥65	2,849	12.4 (5.5–19.4)	10,804	47.2 (28.5–65.8)	—§	—§	14,922	65.1 (47.6–82.7)	52,892	230.9 (175.2–286.6)	53,666	234.4 (178.2–290.3)	0.95	86.9
**Race/Ethnicity** ¶														
White	49,020	48.2 (36.1–60.2)	171,453	167.5 (114.2–222.8)	114,902	112.9 (76.1–149.7)	162,788	160.0 (114.6–205.4)	483,342	475.1 (352.6–597.6)	609,368	598.9 (452.7–745.2)	1.19	91.5†
Black	7,314	35.5 (1.5–69.4)	17,204	83.4 (27.8–139.1)	83,460	404.7 (156.8–652.5)	19,531	94.7 (43.7–145.7)	84,077	407.6 (205.3–610.0)	178,943	867.6 (413.7–1,321.5)	1.79	34.3
Other and unknown race	6,921	71.1 (32.1–110.0)	13,948	143.2 (88.9–197.5)	16,071	165.0 (96.0–234.0)	12,147	124.7 (75.8–173.6)	54,575	560.3 (356.2–764.4)	77,865	799.5 (495.3–1,103.7)	1.33	−36.2
Hispanic	3,896	15.7 (7.2–24.1)	8,733	35.1 (17.4–52.8)	16,625	66.9 (24.4–109.4)	8,952	36.0 (18.1–53.9)	50,055	201.4 (99.9–302.8)	77,190	310.5 (148.2–472.8)	1.80	—§

**Abbreviations:** CI = confidence interval; M:F = male to female.

* Per 100,000 population.

† Significant to at least p<0.05.

§ Numbers and rates are small and might be unstable.

¶ Persons identified as Hispanic might be of any race. Persons identified as any of the other categories were non-Hispanic.

## Footnotes

* Information about bridged-race estimates is available at http://wonder.cdc.gov/wonder/help/mcd.html.

† Information about DAWN files is available at http://www.icpsr.umich.edu/icpsrweb/SAMHDA.
